# Facts and Recommendations regarding When Medical Institutions Report Potential Abuse to Child Guidance Centers: A Cross-Sectional Study

**DOI:** 10.3390/pediatric14040056

**Published:** 2022-11-02

**Authors:** Mio Urade, Misao Fujita, Atsushi Tsuchiya, Katsumi Mori, Eisuke Nakazawa, Yoshiyuki Takimoto, Akira Akabayashi

**Affiliations:** 1 Department of Biomedical Ethics, Faculty of Medicine, University of Tokyo, 7-3-1 Hongo, Bunkyo-ku, Tokyo 113-0033, Japan; 2 Uehiro Research Division for iPS Cell Ethics, Center for iPS Cell Research and Application, Kyoto University, 53 Shogoin Kawahara-cho, Sakyo-ku, Kyoto 606-8507, Japan; 3 Graduate School of Sociology, Kansai University, 3-3-35 Yamate-cho, Osaka 564-8680, Japan; 4 Division of Medical Ethics, School of Medicine, New York University, 227 East 30th Street, New York, NY 10016, USA

**Keywords:** child abuse, notification to family, Japan, fact-finding, cross-sectional study

## Abstract

Background: Medical institutions are required to report suspected cases of child abuse to administrative agencies, such as child guidance centers in Japan. It is left to the discretion of the medical institutions whether to notify the family of the child or the center. However, it is unclear what kinds of measures are being taken to ensure a robust policy of notification versus non-notification and how notifying the family will affect the child. Methods: An unregistered questionnaire survey on reporting suspected child abuse cases to child consultation centers and notifying families was conducted by mail across 518 pediatric specialist training facility hospitals designated by the Japanese Pediatric Society. Results: Responses were received from 323 facilities (62.4% response rate), of which 5 facilities were excluded because of incomplete responses. Therefore, in all, 318 facilities were included in the analysis. The results showed that 59.8% of the facilities had a policy of notifying the family, 33.7% said the decision varies from case to case, and 6.6% did not have a policy of notifying the family. The facilities that had a policy of either notifying or not notifying the family were less likely to experience problems than those with a policy of deciding on a case-by-case basis. The proportion of cases in which some problems occurred was higher in the cases where families were notified than in the cases where they were not, with 51.4% of the children experiencing worsening of relationships with family members. In the cases where the families were not notified, the children were twice as likely to experience further abuse than in cases where the families were notified. Conclusion: Problems arise in the case of both notification and non-notification. It is necessary to examine background factors and specific methods of notification in the cases where problems arise.

## 1. Introduction

Child abuse has apparently increased because of social distancing policies that were put into place to deal with the coronavirus disease 2019 (COVID-19) pandemic [1,2]. According to recent reports, in Japan, 2219 children were victims of child abuse in 2021 (cumulative total based on police arrests), with the number increasing for 17 consecutive years [3,4]. Child abuse represents an act of physical abuse, sexual abuse, neglect, or psychological abuse committed by a parent or a guardian against a child in their care [5]. The Convention on the Rights of the Child, adopted at the 44th United Nations General Assembly [6,7], calls on States Parties to the Convention to take institutional measures against child abuse. In Japan, professionals (medical personnel, teachers, childcare workers, etc.) who are in frequent contact with children on a daily basis are obligated to notify the authorities when they suspect child abuse [5,8].

One of the problems that medical institutions face in dealing with child abuse is notifying families. When medical institutions report cases of child abuse to administrative agencies, such as child guidance centers (CGCs), they are faced with the choice of whether to notify the child’s family (hereafter, the term “report” refers to reporting the suspected abuse to the CGC and “notification” refers to informing the family of the child in the case of suspected abuse). In Japan, there are no laws or guidelines for medical institutions to notify family members. Therefore, each medical institution is required to take its own actions.

Prior research on notification has taken three positions: (1) health care providers should not notify the family, (2) they should notify the family, and (3) they should decide on a case-by-case basis. The main reason for not notifying the family about the suspected child abuse is that notifying the family may place the child directly in a life-threatening situation [9]. Furthermore, notification may result in deterioration of the relationship between the family and the health care provider, which may affect the treatment of the affected child [10]. On the contrary, *Nelson Textbook of Pediatrics*, which is widely used, explicitly states that the family should be notified so trust can be built between the health care provider and the family [11]. Other reasons given so far for notification include the physician’s role in terms of integrity in openly informing the family of suspected abuse [12]. The third position, which says that a decision should be made on a case-by-case basis, argues that decisions about notification should be based on an assessment of the child’s risk of further harm [13,14]. The three patterns of notification contain two different problem axes: the first axis is the advantages and disadvantages of notification; the second axis is whether the decision should be made according to principles or according to circumstances.

Although theoretical considerations regarding notification can be summarized in this way, there is a lack of evidence on exactly what contributes to decisions on notification policies at medical institutions, and this may lead to confusion and conflict. Therefore, this study aimed to investigate the current handling of family notification at medical institutions in Japan in order to contribute to decisions on notification policies at these institutions, clarifying their impact in the medical setting.

## 2. Methods

### 2.1. Facilities

The survey covered all 518 hospitals designated by the Japanese Society of Pediatrics as training facilities for pediatric specialists.

### 2.2. Procedure

Self-administered questionnaires were mailed to the chief pediatricians in pediatric specialist training facility hospitals designated by the Japanese Pediatric Society. At each facility, the pediatrician-in-chief was asked to select one person at that facility familiar with the response to child abuse (hereafter, referred to as the “respondent”), and the respondent answered the questionnaire in terms of the situation at each facility, not in terms of their personal experiences.

### 2.3. Items

#### 2.3.1. Basic Attributes of Facilities

Responding facilities were asked about basic attributes, such as hospital type, number of beds, number of pediatric beds, and experience in reporting child abuse. They were also asked about the number of reports and notifications per year, as well as whether they had an in-house manual or guidelines. Respondents were asked about their age, gender, job title, years of experience in their job title, role in responding to child abuse in the hospital, and years of experience in responding to child abuse.

#### 2.3.2. Notification Policy

Regarding the current policy for handling notices, the respondents were asked to select one of the following options: “notify,” “varies from case to case,” or “do not notify.”

#### 2.3.3. Reason for Notification Policy

On the basis of prior literature, the first author (U.M.) selected 14 items as reasons for the notification response policy and assessed them on a four-point scale [9,13,14]. Their validity was determined through several rounds of review together with the co-authors.

#### 2.3.4. Effects of the Notification

The respondents were asked to choose from a list of seven examples of actual problems they have experienced in cases where they notified or did not notify. Through an open-ended question, the respondents were also asked to provide details of their experiences. On a four-point scale, they were asked to estimate the psychological burden of dealing with family members.

### 2.4. Analysis

For each response, the results were tabulated by the group. The reasons for the notification policy were combined into two categories, “true” and “not true,” on the four-point scale.

The McNemar test of exact probability was used to compare the percentage of facilities that had experienced some problems on notifying and not notifying. Among facilities responding that they had notified the families, we compared through an exact probability χ-square test the percentage of facilities that had experienced some problems as a result of following the notification policy. In addition, we tabulated the experience related to each of the notification policies. To examine the timing of notification, we conducted a survey of the number of facilities that had experienced some difficulties as a result of following a notification policy. We also compared the χ-square tests where the facilities had experienced some difficulties when notifying before reporting and when notifying after reporting.

SAS University Edition was used for the above statistical analysis, and the statistical significance level was set at 5%.

### 2.5. Ethical Considerations

This study was approved by the Ethics Committee of the University of Tokyo School of Medicine (Approval No. 10220).

## 3. Results

### 3.1. Response Rate and Basic Attributes of Responding Facilities and Respondents

The collection rate was 62.4% (number collected/number distributed = 323/518). Of these, 318 facilities were included in the analysis after the responses of 5 facilities that did not respond to the basic attributes or notification policies were excluded. Regarding the bed size, about one-third of the facilities had 250–499 beds and about 40% had 500–749 beds (Table 1). In terms of the number of pediatric beds, 33.6% had 24 or fewer beds and 48.4% had 25–49 beds. Around 88.8% of the facilities indicated that they had given reports to the CGC, and 11.2% of the facilities indicated that they had never given reports to the CGC.

The average age of the respondents was 49.3 years, the average number of years in the profession was 23.7 years, and the average number of years dealing with child abuse cases was 9.7 years (Table 2). Approximately 80% of the respondents were male, and 93.4% were physicians. About 70% of the respondents held some kind of position that dealt with cases of child abuse.

### 3.2. Response Rate and Basic Attributes of Responding Facilities and Respondents

Regarding the policy of notifying the family after reporting suspected abuse to the CGC, the response of 59.8% of the facilities was “basically notify,” that of 33.7% of the facilities was “vary from case to case,” and that of 6.6% of the facilities was “basically do not notify” (Table 3).

Regarding the number of notifications between April 2012 and March 2013, the most common was 1–5 cases (54.7%). The highest number of notifications to family members was 1–5 (46.8%). The average number of reports was 3.4, with a maximum of 50 and a minimum of 0. The average number of notifications was 2.9, with a maximum of 38 and a minimum of 0.

### 3.3. Reasons for the Notification Policy

**Figure 1:**
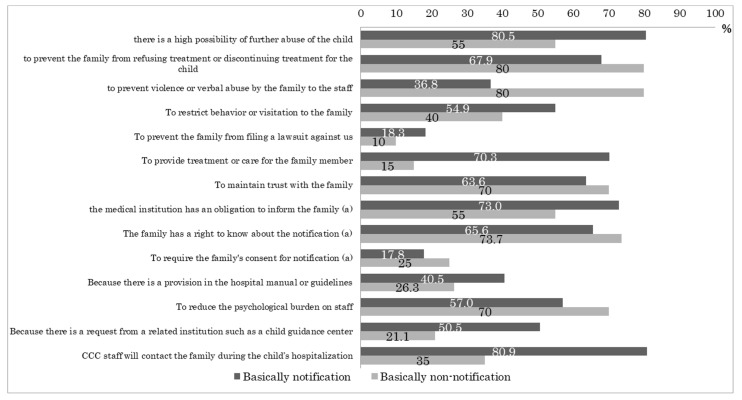
Reasons for the response policy for the notification and non-notification groups. Note: The number for each item = (number of facilities that chose “true” or “somewhat true”)/(number of facilities that answered the entire item). (a) In the non-notification group, the following items are inverted: “the medical institution is not obliged to inform the family,” “the family has no right to know about the notification,” and “the family’s consent is not necessary for the notification.” For facilities that did not choose “true” or “somewhat true” for any of the above items, “true” was taken as the response and the responses were included in the percentages calculated in the table in this figure. Since the items are not the same as those of the notification group, we need to be careful in interpreting the results.

Figure 1 summarizes the percentage of facilities that answered in the affirmative (i.e., with a “yes”) to 14 items obtained from previous studies regarding the reasons for the notification policy for the notification and non-notification groups. The top three reasons for notification in the notification group were “CGC staff will contact the family during the child’s hospitalization” (80.9%), “there is a high possibility of further abuse of the child” (80.5%), and “the medical institution has an obligation to inform the family” (73.0%).

In the non-notification group, the top three reasons for not notifying were “to prevent the family from refusing treatment or discontinuing treatment for the child” (80.0%), “to prevent violence or verbal abuse by the family to the staff” (80.0%), and “family has no right to know about the notification” (73.7%).

### 3.4. Effects of Notification

#### 3.4.1. Experienced Difficulties after Responding to Notifications

Only those facilities with a reporting experience were counted (Table 4). Of the 278 facilities, 176 facilities (63.3%) responded that they had experienced some difficulties as a result of notifying the family in conjunction with the report. Fifty-two facilities (18.7%) responded that they had experienced some difficulties when they reported the matter to the CGC but did not notify the family. The percentage of respondents who experienced some problems was significantly higher when they notified the family than when they did not (*p* = 0.001). The most common problem experienced as a result of notification was “worsening of relationship with family” (at 143 facilities; 51.4%). Even in the case where the family was not notified, the most common problem experienced was “worsening of relationship with family” (at 25 facilities; 9%).

#### 3.4.2. Notification Policy and Experienced Difficulties

Table 5 shows the percentage of facilities that experienced difficulties as a result of following the notification policy.

In the notification group, the most common problem experienced as a result of notification was “worsening of the relationship with the family” (at 79 facilities; 45.9%). In the non-notification group, only two facilities (11.1%) indicated that they had experienced difficulties as a result of not notifying the patient’s family: “the family requested early discharge or transfer” and “the child suffered further abuse.”

In the case-by-case group, the most common problem experienced in the case of notification was “worsening of the relationship with the family” (56 facilities; 64.4%). The most common problem experienced in the case of non-notification was also “deterioration of relationship with family” (13 facilities; 14.9%).

When facilities were notified, the proportion of facilities that experienced any difficulty was significantly higher in the case-by-case notification group than in the notification group (*p* = 0.015). When facilities did not notify the families, the percentage of facilities that experienced some difficulties was significantly higher in the case-by-case non-notification group than in the non-notification group (*p* = 0.05).

#### 3.4.3. Timing of Notification and Experienced Difficulties

For the notification and case-by-case notification groups, the difficulties they faced on notifying families before reporting to the CGC and those they faced on notifying families after reporting to the CGC were compared (Table 6). A significantly larger proportion of facilities experienced some problems when they notified the families after reporting to the CGC. For all seven of the difficulties that occurred with notification, a greater percentage of facilities experienced them on notification after reporting than on notification before reporting.

#### 3.4.4. Psychological Burden of Dealing with Family Members

Among the total respondents, 156 (56.3%) said they “often” and 94 (33.9%) said they “sometimes” experienced psychological burden in dealing with family members. In the notification group, 90 (52.3%) and 66 (38.4%) of the respondents answered “often” and “sometimes,”, respectively; in the case-by-case group, 58 (66.7%) and 24 (27.6%) of the respondents answered “often” and “sometimes,”, respectively; in the non-notification group, 8 (44.4%) and 4 (22.2%) respondents answered “often.”

## 4. Discussion

This study is the first survey in Japan to clarify the actual situation and effects of notifications by medical institutions to families regarding child abuse. The collection rate for this survey was high, at 62.4%, and there was no bias found when the number of questionnaires distributed and collected was compared by hospital type and region. The largest number reports made by medical facilities to the CGC annually was 1–5 cases by 168 facilities (54.7%), with an average number of 3.4 reports/year. The most common annual number of notifications to families was 1–5 at 141 facilities (46.8%), with an average number of 2.9 notifications/year.

### 4.1. Notification Policy and Reasons

With regard to the policy on notification to families, more than half of all facilities (190 facilities; 59.8%) had a policy of “notifying.” More than 80% of the facilities gave one of the following reasons: “CGC staff will contact the family during the child’s hospitalization” and “there is a high possibility of further abuse of the child.” From the reason “there is a high possibility of further abuse of the child,” one can conjecture that notifying the family can deter the family. However, prior literature [15,16,17,18] states that notification should be refrained from when the risk of further abuse is high and the corresponding reason for notification is contrary to the assertion in the prior literature. About half of the facilities cited “because of requests from CGC” as the reason for the notification policy. In a survey of CGCs, 65% of the CGC staff reported that “doctors notify, but do not tell parents of abuse” as a problem [19], suggesting that CGCs expect medical institutions to notify families in order to provide a starting point for intervention by them and other administrative agencies [11].

In our study, a small number of facilities, 21 (6.6%), did not notify. In the non-notifying group, about 80% of the facilities gave the following reasons: “to prevent the family from refusing treatment or discontinuing treatment for the child” and “to prevent violence and verbal abuse by the family toward staff.” Among the non-notification group, facilities seemed to emphasize the treatment of the child, which is the highest priority of the medical institution, and the protection of the staff as the chief reasons for non-notification. In previous literature, risks such as increased danger to the child were pointed out as reasons for not notifying [19], while in this study, there were no indications of violent outbursts against the staff. Compared to international circumstances, where the police are the main contact for reports of child abuse [20], the lack of police and judicial intervention has been pointed out in the response to child abuse in Japan [21]. As a result, violence and other forms of harm to the staff may be the reasons facilities do not notify the family.

A contrasting result between the notified and non-notified groups was found regarding the family’s right to know. In the notification group, 65.6% of the facilities cited the family’s right to know as the reason for their response, and in the principal non-notification group, 73.0% of the facilities responded that the medical institution had an obligation to notify the family. In Japan today, there is a mixture of individualism and family-centeredness. It has been reported that family influence is also significant in medical decision making [22]. It is presumed that family-centeredness will be higher, especially for minor patients. The facilities in the notification group may be more influenced by traditional family-centered medicine, which emphasizes the family’s right to know.

### 4.2. Difficulties Arising with Notification and Non-Notification

A comparison of the difficulties arising with and without notification showed that significantly more problems were experienced when notification was made. In the case of notification, the relationship with the family worsened in more than half of the facilities and the family wished for early discharge or transfer in 30% of the facilities. Although “to prevent the family from refusing or discontinuing the child’s treatment” was the most important reason for notification in the notification group, the results showed that the opposite effect occurred as a result.

The percentage of facilities where children experienced further abuse was higher in the cases where the families were not notified than in the cases where they were. The response that it is better not to notify the family when the risk of further child abuse is high, as pointed out in previous literature [15,16,17,18], suggests that this is not necessarily true for the prevention of further abuse. Although the number of cases of further abuse is small, it is the most important issue to avoid, and it is clear that there are risks to the child’s safety in both cases of notification and non-notification.

Since risks exist for both notification and non-notification, it is desirable to base the decision to notify or not to notify family members on a case-by-case risk assessment for risks arising from the notification. However, in this study, in the case-by-case group, problems occurred significantly more frequently in the case of notification than in the basically notification group and more frequently in the case of non-notification than in the basically non-notification group. This result may suggest the difficulty in assessing risk on a case-by-case basis and in selecting notification behavior according to risk. When there is suspected child abuse, to reduce problems after the notification, it may be more effective for the medical institution to decide in advance whether to notify rather than considering and changing the response for each case. Careful risk assessment and adjustment should be made on the basis of this premise for each individual case.

### 4.3. Difficulties Arising and Timing of Notification

Notifying the family before reporting the matter to the CGC was significantly less likely to cause problems than notification after reporting, indicating that differences in the timing of notification were associated with differences in the rate of problems. The results were consistent with prior literature recommending notification before reporting [12,23]. The cases in which families were notified after the matter was reported to the CGC included cases with a greater degree of family intervention, such as emergency temporary protection of the child [24], which may have resulted in greater family opposition. A detailed investigation is needed to determine whether notifying after reporting itself raises the risk of problems occurring.

### 4.4. Psychological Burden of Dealing with Family Members

Overall, 90% of the respondents found dealing with families to be a burden. Among these, the psychological burden was particularly prevalent in facilities that responded on a case-by-case basis. Deciding on a policy for notifying families as a facility may contribute to reducing the burden on the staff who respond to the cases.

### 4.5. Limitations

There are several limitations to this study. (1) The survey was conducted among pediatric specialist training facility hospitals of the Japanese Pediatric Society. Therefore, relatively large medical institutions were included in the study. Among the medical institutions in Japan that responded to this survey, 18.1% had 50 or more beds, while 7.2% had 50 or more pediatric beds [25]. Therefore, the response to family notification may also have been handled at relatively large medical institutions and the response to family notification may differ at small- and medium-sized medical institutions. In the future, it will be necessary to conduct surveys of small- and medium-sized medical institutions, as well as surveys of cases of suspected child abuse not only in inpatient wards but also in outpatient wards, in order to grasp the overall picture of family notification handled by medical institutions. (2) Although this survey clarified the policy for notifying family members, the reasons for the policy, and its effects, it did not clarify the circumstances that led to each medical institution’s policy for notifying family members. A detailed investigation of the background to the current policy is needed in the future. (3) There are limitations inherent to the way in which the questions were asked about problems that actually occurred in the medical field. The survey was not about the number of problems that occurred but rather about whether respondents experienced problems. Therefore, there may be a gap between the number of problems and the actual cases. In the future, to analyze and define the details of the problems that occurred, it will be necessary to analyze the background of each case to determine how many of them actually occurred. Furthermore, it will be necessary to clarify which cases are more likely to cause problems and to identify the factors affecting the families and children, as well as to investigate and study the method of notification. (4) Although the survey results were old and may not fully reflect the current situation, we are preparing a follow-up survey, as of 2022, due to the increase in child abuse after the COVID-19 pandemic. We believe that this paper is significant in terms of providing basic data for making such comparisons. To examine the possibility of creating criteria for cases where the families should be notified and the case where they should not, and to develop clearer and more specific guidelines, in future studies, it is necessary to clarify the factors that cause problems when families are notified.

## 5. Conclusions

More than half of the facilities decided to notify the families of suspected victims of child abuse as well as to report the matter to the CGC. The facilities that responded on a case-by-case basis faced a higher rate of difficulties than those that decided to respond in principle. The results of this survey suggest that it is desirable for medical facilities to establish a policy of either informing or not informing the family and to consider measures to deal with the problems that are likely to occur in each case.

## Figures and Tables

**Table 1:** Basic attributes of responding facilities (n = 318).

	n (%)
Hospital Type	
Children’s hospital	14 (4.4)
University hospital	81 (25.6)
General hospital	217 (68.7)
Other	4 (1.3)
Hospital Location	
Hokkaido	18 (5.7)
Tohoku	18 (5.7)
Kanto	87 (27.4)
Chubu	68 (21.5)
Kinki	69 (21.8)
Chugoku	14 (4.4)
Shikoku	12 (3.8)
Kyushu	31 (9.8)
Total Number of Beds	
≤249 beds	20 (6.5)
250–499 beds	105 (34.3)
500–749 beds	120 (39.2)
750–999 beds	36 (11.8)
1000 beds	25 (8.2)
Pediatric Beds	
≤24 beds	104 (33.6)
25–49 beds	150 (48.4)
50–74 beds	39 (12.6)
75–99 beds	5 (1.6)
100 beds	12 (3.9)
Report Experience	
Yes	278 (88.8)
No experience	35 (11.2)

Note: Percentages were calculated excluding 2 respondents who did not respond to the question about hospital type, 12 respondents who did not respond to the question about the total number of beds, 8 respondents who did not respond to the question about the number of pediatric beds, and 5 respondents who did not respond to the question about the hospital location and report experience.

**Table 2:** Basic attributes of respondents (n = 318).

	n (%)
Gender	
Male	252 (79.5)
Female	65 (20.5)
Occupation	
Doctor	296 (93.4)
Nurse	18 (5.7)
Social worker	2 (0.6)
Clinical psychologist	1 (0.3)
Position Related to Child Abuse Cases ^a^	
Yes	217 (70.2)
Not employed	92 (29.8)
	Average (min., max.)
Age	49.3 (28, 66)
Years of experience in the profession	23.7 (2, 41)
Years involved in dealing with child abuse cases child abuse child abuse	9.7 (0, 37)

Note: The percentages of respondents who answered to “Position Related to Child Abuse Cases” were calculated excluding nine respondents who did not answer. Gender and occupation were calculated excluding one non-responder. ^a^ Based on the analysis of free answers to the question “Your role in responding to child abuse” in the questionnaire.

**Table 3:** Notification policy and number of reports and notifications (April 2012–March 2013; n = 318).

	n (%)
Notification Policy	
Notification	190 (59.8)
Varies from case to case	107 (33.7)
No notification	21 (6.6)
Number of Cases Notified in a Year	
0 cases	94 (30.6)
1–5 cases	168 (54.7)
6–10 cases	31 (10.1)
11–15	8 (2.6)
16 cases	6 (2.0)
Number of Notices per Year	
0	122 (40.5)
1–5	141 (46.8)
6–10	25 (8.3)
11–15 notices	7 (2.3)
16 notices	6 (2.0)

Note: Percentages were calculated excluding 11 respondents who did not respond to the question about the number of reports, 17 respondents who did not respond to the question about the number of notifications, and 45 respondents who did not respond to the question about the existence of hospital response manuals or hospital guidelines.

**Table 4:** Difficulty experienced with and without notification, n (%).

	In Case of Notificationn (%)	In Case of Non-Notificationn (%)	*p*-Value *
Overall	278 (100)	278 (100)	
Experienced some kind of problem ^†^	176 (63.3)	52 (18.7)	<001
Content of Problems Experienced (multiple responses allowed)			
Relationship with family deteriorated	143 (51.4)	25 (9.0)	
Family requested early discharge or transfer	80 (28.8)	14 (5.0)	
Family refused treatment for the child	39 (14.0)	9 (3.2)	
Family was violent toward the staff or harmed the staff in some other way	38 (13.7)	8 (2.9)	
The family removed or attempted to remove the child	38 (13.7)	6 (2.2)	
The child suffered further abuse	10 (3.6)	19 (6.8)	
The family filed a lawsuit	7 (2.5)	3 (1.1)	

Note: Only the facilities that responded that they had reported the cases to the CGC or had notified the families were included in the sample (n = 278). * McNemar test (*p*-value is based on the exact probability). ^†^ When the option chosen was “experienced some kind of problem,” the respondents were defined as “experienced” if they answered that they had experienced at least one of the seven problems.

**Table 5:** Notification policy and difficulty experienced, n (%).

	Notification Group	Case-by-Case group Notification	*p*-Value *	Non-Notification Group	Case-by-Case group Non-notification	*p*-Value *
Overall	172 (100)	87 (100)		18 (100)	87 (100)	
Experienced some kind of problem ^†^	102 (59.3)	65 (74.7)	0.015	2 (11.1)	30 (34.5)	0.050
Content of Problems Experienced (multiple responses allowed)						
Relationship with family deteriorated	79 (45.9)	56 (64.4)		0 (0)	13 (14.9)	
Family requested early discharge or transfer	43 (25.0)	31 (35.6)		1 (5.6)	9 (10.3)	
Family refused treatment for the child	15 (8.7)	18 (20.7)		0 (0)	4 (4.6)	
Family was violent toward the staff or harmed the staff in some other way	22 (12.8)	13 (14.9)		0 (0)	4 (4.6)	
The family removed or attempted to remove the child	17 (9.8)	18 (20.7)		0 (0)	4 (4.6)	
The child suffered further abuse	6 (3.5)	2 (2.3)		1 (5.6)	10 (11.5)	
The family filed a lawsuit	3 (1.7)	3 (3.5)		0 (0)	1 (1.2)	

Note: Only the facilities that responded that they had reported the cases to the CGC or had notified the families were included in the sample. * χ-square test (*p*-value is based on the exact probability). ^†^ When the option chosen was “experienced some kind of problem,” the respondents were defined as “experienced” if they answered that they had experienced at least one of the seven problems.

**Table 6:** Difficulties arising and timing of notification.

	Pre-Report n (%)	Post-Report n (%)	*p*-Value *
Overall	73 (100)	67 (100)	
Experienced some kind of problem ^†^	40 (54.8)	52 (77.6)	0.005
Content of Problems Experienced (multiple responses allowed)			
Relationship with family deteriorated	30 (41.1)	42 (62.7)	
Family requested early discharge or transfer	16 (21.9)	20 (29.9)	
Family refused treatment for the child	4 (5.5)	6 (9.0)	
Family was violent toward the staff or harmed the staff in some other way	6 (8.2)	11 (16.4)	
The family removed or attempted to remove the child	3 (4.1)	11 (16.4)	
The child suffered further abuse	1 (1.4)	3 (4.5)	
The family filed a lawsuit	1 (1.4)	1 (1.5)	

Note: Only the notification group and the case-by-case group notifications were included in the sample of facilities that responded that they had reported the cases. To compare the results before and after the notification, we excluded those who answered that they chose to notify the family on a case-by-case basis. * χ-square test (p-value is based on the exact probability). ^†^ When the option chosen was “experienced some kind of problem,” the respondents were defined as “experienced” if they answered that they had experienced at least one of the seven problems.

## Data Availability

The datasets analyzed in the current study are available from the corresponding author upon reasonable request.
